# ASO Author Reflections: Non-radioactive Sentinel Node Localization with Superparamagnetic Iron Oxide in Clinically Node-Negative Breast Cancer Patients: A Possibility for Improvement of the Care Pathway

**DOI:** 10.1245/s10434-020-09325-5

**Published:** 2020-11-10

**Authors:** Maria Margarete Karsten, Sina Shams, Friedrich Kühn

**Affiliations:** Department of Gynecology, Corporate Member of Freie Universität Berlin, Humboldt-Universität zu Berlin and Berlin Institute of Health, Charité–Universitätsmedizin Berlin, Berlin, Germany

## Past

Sentinel node biopsy (SNB) was first established in the therapy of early-stage melanoma,^1^ then subsequently adopted for other oncologic entities. Currently, it is a pivotal part of the staging and therapy for breast cancer patients. In this context, various agents for sentinel lymph node localization have proven suitable for reaching high detection rates and low false-negative rates.^2^ Radioactive localization using technetium^99^ (Tc^99^) in combination with blue dye or as a single tracer has long been considered the “gold standard.” However due to the short half-life of technetium, injections need to be scheduled close to surgery. Radiation protection measures, and in Germany additional lymphoscintigraphy, are mandatory and extend the preparation time. A more flexible schedule and thus an increase in patient comfort might be achieved by using superparamagnetic iron oxide (SPIO). Proven equivalent to Tc^99^ for primary SNB by multiple meta-analyses,^2^, ^3^ SPIO can be administered up to 7 days before surgery.

## Present

This study enrolled 59 patients at Charité–Universitätsmedizin Berlin to investigate the impact of SPIO use for SNB on the care process, reimbursement, surgical time, and patient comfort compared with ^99^Tc.^4^ The preoperative preparation time was significantly shorter for the SPIO group (SPIO, 5.4 ± 1.3 min vs TC^99^, 82 ± 20 min; *p* < 0.0001), even with omission of the time spent for lymphoscintigraphy (TC^99^, 54.4 ± 13.6 min; *p* < 0.0001). Also, the duration of the sentinel lymph node extraction was slightly shorter (SPIO, 5 min [range, 3–15 min] vs TC^99^, 10 min [range, 7–15 min]; *p* = 0.151). With SPIO, the duration of the whole SNB procedure also was shorter (SPIO, 9 min [range, 4–15 min] vs TC^99^, 10 min [range, 7–15 min]; *p *= 0.412) despite the fact that the iron-based system was a new method at our institution. Concerning pain assessment and reimbursement, the study could not detect any significant differences between the two groups. The study was limited by its small sample size, non-randomized group allocation, and variation in surgical procedures (mastectomy and breast-conserving surgery). In hindsight, detailed patient-reported experience measurements would have been favorable for examination of patient comfort.

## Future

The aforementioned findings show that SPIO-based sentinel-node localization facilitates a shortened preoperative preparation time and has no negative impact on reimbursement in the German health care setting. Due to a multitude of reasons such as lack of health care professionals, decreased financial resources, and development of even more complex therapy regimens, specialized oncologic care probably will be provided in fewer but larger centers, thus increasing travel time for patients and making scheduling of procedures even more critical. Flexible and less time-consuming alternatives to radioactive sentinel node-marking, such as the SPIO-guided approach, are necessary, especially in rural areas, where nuclear medicine facilities usually are scarce. Further studies should investigate the safety of SPIO for SNB after neoadjuvant chemotherapy (NACT). Currently, dual tracing with TC^99^ and blue dye is recommended in this context. In addition, the SPIO application method and dose for patients in need of postoperative magnetic resonance imaging (MRI) still need to be examined.^5^

## References

[1] Morton DL, Wen DR, Wong JH (1992). Technical details of intraoperative lymphatic mapping for early-stage melanoma. Arch Surg..

[2] Mok CW, Tan SM, Zheng Q, Shi L (2019). Network meta-analysis of novel and conventional sentinel lymph node biopsy techniques in breast cancer. BJS Open..

[3] Karakatsanis A, Christiansen PM, Fischer L (2016). The Nordic SentiMag trial: a comparison of super paramagnetic iron oxide (SPIO) nanoparticles versus Tc^99^ and patent blue in the detection of sentinel node (SN) in patients with breast cancer and a meta-analysis of earlier studies. Breast Cancer Res Treat..

[4] Shams S, Lippold K, Blohmer JU, Roehle R, Kühn F, Karsten MM. A pilot study evaluating the effects of Magtrace for sentinel node biopsy in breast cancer patients regarding care process optimization, reimbursement, surgical time, and patient comfort compared to standard technetium^99^. *Ann Surg Oncol.* 2020. 10.1245/s10434-020-09280-1. 10.1245/s10434-020-09280-1 PMC8119277 33263157

[5] Karakatsanis A, Obondo C, Abdsaleh S, Hersi A-F, Eriksson S, Wärnberg F (2018). Optimisation of breast MRI compatibility after sentinel node biopsy with paramagnetic tracers. Eur J Surg Oncol..

